# *Clostridium difficile* colitis – a serious current problem

**DOI:** 10.1186/1471-2334-13-S1-O20

**Published:** 2013-12-16

**Authors:** Irina Magdalena Dumitru, Eugen Dumitru, Roxana Carmen Cernat, Andra Elena Petcu, Carmen Ilie Șerban, Sorin Rugină

**Affiliations:** 1 Ovidius University, Constanța, Romania; 2 Clinical Hospital of Infectious Diseases, Constanța, Romania

## Background

*Clostridium difficile* is currently considered a significant cause of nosocomial infection and is associated with increasing morbidity and mortality. The probability of colonization of hospitalized patients increases with the length of their hospital stay and depends on the local epidemiologic situation.

The study aimed to evaluate the epidemiological, clinical and treatment features of *Clostridium difficile* colitis (CDC), and the relapse associated risk factors in the Infectious Diseases Clinic and Gastroenterology Clinic.

## Methods

Prospective study of cases admitted to our clinic with CDC in the last year. The following parameters were analyzed: age, sex, comorbidities, recent hospitalizations, recent surgery, use of antibiotics, use of proton pump inhibitors (PPIs), clinical form of the disease, methods of diagnosis and therapeutic response.

## Results

In the two Clinics, 42 patients were diagnosed and treated in the last year (14 times more than during 2011-2012). More than half of the cases had severe clinical forms (pancolitis). We recorded no case of toxic megacolon. All patients reported use of antibiotics, most frequently fluoroquinolones and cephalosporins (48% and 34%). 68% of patients had history of prolonged hospitalizations in orthopedics, surgery, oncology or hematology clinics and 82% of patients had comorbidities (leukemia, cancer, liver cirrhosis). The toxin (A and B) test was positive in only half of cases, in all cases the diagnosis was confirmed by sigmoidoscopy. Metronidazole, vancomycin and rifaximin were administered in 68% of cases, intravenous metronidazole, ertapenem and rifaximin in 10% of cases, metronidazole orally and rifaximin in 10% of cases and only 12% of cases responded favorably to metronidazole alone. We recorded relapses in 9 patients (21.4%). Relapse associated risk factors were: malignant diseases, inflammatory bowel disease, IPP treatment, colonic resection, immunosuppressive therapy and absence of rifaximin regimen.

## Conclusion

Interdisciplinary collaboration is vital for limiting the development of this very serious, often fatal disease. Rational use of antibiotic therapy is essential, particularly in an environment contaminated by spores of *Clostridium difficile*. We need to examine the possibilities of using vaccines to combat infection due to *Clostridium difficile* in real life and clinical trials.

